# The protective mechanism of *Naja Naja atra* venom on diabetic kidney disease

**DOI:** 10.1590/1678-9199-JVATITD-2023-0037

**Published:** 2023-12-08

**Authors:** HongYu Lu, YaJuan Wu, Yan Xie, XiaoWei Li, Xian Ji, TianHui Jiang, XiaoXian Pei, ZhuYa Zhou

**Affiliations:** 1 Department of Psychosomatic Medicine, The Fourth People's Hospital of Zhangjiagang, Zhangjiagang, Jiangsu, China.; 2 The First Affiliated Hospital of Soochow University, Suzhou, Jiangsu, China.

**Keywords:** Diabetic kidney disease, cobra α-neurotoxin, *Naja Naja atra* venom, Urine protein

## Abstract

**Background:**

Diabetic kidney disease (DKD) is a serious microvascular complication of diabetes that affects both type 1 and type 2 diabetes patients at a high incidence rate. *Naja Naja atra* venom (NNAV) has been shown to have protective effects and improved renal function in diabetic rats. However, its mechanism of action is still unclear. This study aims to unravel the effectiveness and mechanisms of NNAV on DKD.

**Methods:**

We conducted in vitro experiments in which Human Kidney-2 (HK-2) cells were stimulated with high glucose, and exposed to varying concentrations of NNAV. Cell morphology, as well as α-SMA, TGF-β1, and E-cadherin levels, were analyzed using immunofluorescence and western blot. *In vivo* experiments involved a diabetic rat model, where varying concentrations of cobra α-neurotoxin (CTX) were administrated via gastric treatment. We observed and noted pathomorphological changes, measured biochemical and oxidative stress indices, and used western blot to assess podocin and nephrin levels.

**Results:**

High glucose levels can induce a decrease in E-cadherin expression and an increase in α-SMA and transforming growth factor-β1 (TGF-β1) expression in HK-2 cells. NNAV can inhibit the transdifferentiation of HK-2 cells to myofibroblast (MyoF) in a high glucose environment and reduce the expression of TGF-β1. Cobra α-neurotoxin (CTX) can reduce urine protein in diabetes model rats at an early stage, which is dose-independent and has a time application range. CTX can regulate the expression of nephrin and podocin.

**Conclusion:**

The present study indicates that CTX and NNAV attenuate STZ and high glucose-induced DKD. Its mechanisms of action are associated with inhibiting oxidative stress and TEMT. The study suggests that NNAV and CTX might be a potential therapeutic drug for treating DKD.

## Introduction

Diabetic kidney disease (DKD) is a grave microvascular complication of diabetes mellitus [1]. The disease is characterized by glomerular hypertrophy, thickened basement membrane, an augmented matrix in the mesangial area, and thickening and transparency of the renal vascular system, ultimately leading to nodular glomerulosclerosis, also called Kimmelstiel-Wilson lesions [2]. The initial clinical indication of DKD is the excretion of microalbumin in urine, followed by clinical albuminuria, and eventually chronic kidney disease [3]. Epidemiologic data reveal that approximately 30-40% of patients with T1DM and T2DM develop DKD, and about 50% of them can progress to end-stage renal disease (ESRD) [4]. As the incidence rate and mortality of DKD continue to rise, it poses a serious threat to human health and adversely affects the quality of life of affected individuals. Consequently, DKD has emerged as a worldwide medical research topic.

The cobra α-neurotoxin, derived from the cobra family, is the primary chemical component of NNAV. Compared with other components in cobra venom, it has a smaller molecular weight, ranging from 6 to 8 kD [5]. Research has found that the median lethal dose (LD_50_) of NNAV in mice through oral administration and intravenous injection is 102.3 mg/kg and 0.624 mg/kg, respectively, with a 95% range of 84.9-123.3 and 0.572-0.680 mg/kg, respectively [6]. According to long-term toxicity studies of CTX on rats, high doses (1000 µg/kg) have certain toxic effects on the liver and lungs, which are reversible. Low doses (250 µg/kg) have no toxic effects, and it is feasible to use them as drugs in the body [7]. Neurotoxic components separated from cobra venom may have been used to treat acute and chronic pain diseases, herpes zoster, rheumatoid arthritis, and advanced tumors, and have demonstrated satisfactory analgesic effects [8]. A new type of non-addictive analgesic drug has been developed for analgesia, anesthesia, and drug abstinence, based on the nature of neurotoxin blocking the nerve conduction process [9]. In our comprehensive prior study, we investigated the multifaceted therapeutic capacity of NNNAV on Streptozotocin-induced DKD in Sprague Dawley rats. Over a 12-week trial, variant dosage groups were assessed. Notably, NNAV's effectiveness peaked at 90 µg/kg/day, demonstrating significant improvement in serum glucose, lipid profiles, kidney function indicators viz., microalbumin, N-acetyl-β-glucosaminidase, and cystatin C, and renal oxidative stress markers. Concurrently, the albumin and high-density lipoprotein-cholesterol levels were enhanced. Histopathological observations showcased improved renal structure with reduced glomerular hypertrophy and lesser expression of transforming growth factor-β1 and nuclear factor-κB. The data reinforce NNAV's prospects as a therapeutic intervention for DKD, underscoring its anti-inflammatory, antioxidant, and glucose-lowering attributes, coupled with its lipid regulatory and renal function enhancement capabilities [10]. The clinical application value of NNAV is increasingly recognized.

It has been discovered that under pathological conditions, renal tubular epithelial cells undergo transdifferentiation into MyoF, a process known as tubular epithelial myofibroblast transdifferentiation or epithelial-mesenchymal cell transformation [11]. TEMT plays a crucial role in the development of chronic kidney diseases. *In vitro* and *in vivo* models, several factors can induce TEMT, including transforming growth factor TGF-β1, epithelial growth factor, basic fibroblast growth factor, connective tissue growth factor, interleukin-1, and angiotensin-II [12]. In the hyperglycemic environment, Transforming growth factor-β1 (TGF-β1) is highly expressed in renal tubular cells, currently known as the most potent growth factor for inducing TEMT [13]. It can produce a variety of biological effects through different signal transduction pathways, including hyperglycemia, advanced glycation end products (AGEs), activation of PKC, vasoactive substances such as endothelin and angiotensin II, glucosamine, and MAPK. Oxidative stress promotes the expression of TGF-β1.TGF-β1 plays a central role in renal tubulointerstitial fibrosis by stimulating the expression of the implicated protein, type I and IV collagen, and laminin, inhibiting matrix degradation proteases, and activating protease inhibitors.

Proteinuria is the most common clinical manifestation of DKD. It is not only a crucial biomarker of DKD but also a key factor in promoting the rapid progression of the disease [14]. Recently, some clinical studies and animal experiments have confirmed that the occurrence of albuminuria is still closely related to podocyte damage, including podocyte hypertrophy, podocyte process loss, podocyte separation, apoptosis from the basement membrane, and possibly epithelial-mesenchymal transformation. Podocyte injury as a common trigger can lead to various forms of glomerulopathy, including DKD. The podocyte-related proteins that play an essential role are mainly nephrin and podocin. Nephrin is specifically expressed in the hiatal septum. It is the coding product of the Finnish congenital nephrotic syndrome pathogenic gene NPHS1 gene. Its clinical manifestation is that the fetus can have a large amount of proteinuria in utero, and it is a nephrotic syndrome at birth [15]. It was found that whether the expression of podocin was increased or decreased led to a large amount of albuminuria, which was related to the formation of albuminuria [16]. The experiment suggests that nephrin and podocin show parallel changes both at the protein level and mRNA expression, indicating that nephrin, as a signal protein, is closely related to podocin. Only when they express or secrete together can they transmit signals to the intracellular CD2 binding protein, and then connect with actin through actin 4 to keep the foot process in a normal functional state [17]. This study focuses on revealing the possible mechanisms of NNAV as a therapeutic agent for DKD. Utilizing a rat model of diabetes and Human Kidney-2 (HK-2) cells for *in vitro* studies simulating the high glucose environment seen in diabetic conditions, we explore whether NNAV can mitigate DKD and uncover the prospective molecular mechanism involved.

## Methods

### Ethics statement

Approval was obtained from the Office of Scientific Research Management of Soochow University for the use of animals in this study. All animals were housed in the Laboratory Animal Center of Soochow University and were provided with standard chow pellets and ad libitum access to water. Strictly abide by the national laboratory animal-related laws, regulations, and standards, including but not limited to the “Guidelines for ethical review of the welfare of laboratory animals” (GB/T35892-2018), IGP 2012 and IAVE Guidelines 2010.

### Animals 

Male Wistar rats, aged seven weeks and weighing 160-200g, were purchased from Shanghai SLAC Laboratory Experimental Animals and determined to be specific pathogen-free (SPF). All animals were housed in a controlled environment with temperatures between 21 °C and 25 °C, a 12-hour light/dark cycle, and 50% to 70% humidity. After a one-week acclimation period, animals with blood glucose levels greater than 120 mg/ml were excluded from the experiment.

### Experimental protocol

The study employed 60 male Wistar rats, which were randomly assigned to one of six groups of ten animals each: (1) Normal control group (NC, n = 10); (2) Streptozotocin group (STZ, n = 10); (3) STZ+10 µg/kg/day CTX group (STZ+10 CTX, n = 10); (4) STZ+20 µg/kg/day CTX group (STZ+20 CTX, n = 10); (5) STZ+40 µg/kg/day CTX group (STZ+40 CTX, n = 10); (6) STZ+tripterygium group (STZ+tripterygium, n = 10). DKD was induced by intraperitoneal injection of STZ at a dose of 55 mg/kg in 0.1 mmol/L Na-citrate (pH 4.5) after a 10-hour fast. Rats that showed blood glucose levels over 16.7 mmol/L after an 8-hour fast were considered to have diabetes. A urine glucose level of 3+ to 4+ was indicative of successful DKD. The STZ+tripterygium group received 10 mg/kg/day of tripterygium once a day for 14 weeks by oral gavage, while the STZ+CTX groups (10 μg/kg, 20 μg/kg, 40 μg/kg/d) received neurotoxin once a day for 14 weeks by oral gavage. The remaining two groups were administered the same amount of saline. No insulin was administered throughout the study to control blood glucose levels.

### Urine, serum, and renal tissue collection and detection

Two days before the sacrifice, all rats in each group were placed in metabolic cages to collect 24-hour urine samples. The urine protein content was detected using Coomassie Brilliant Blue. During the study, urine volume and protein levels were measured at 2, 6, 10, and 14 weeks to assess changes over time. At the end of the study, serum creatinine, urea nitrogen, triglyceride, and cholesterol levels were measured using a biochemical analyzer.

Rats were euthanized with 2% pentobarbital sodium, and approximately 4 ml of blood was collected from the abdominal aorta after fixation. Serum samples were collected and stored at -80 ℃ for further analysis. Kidney and pancreas tissues were collected from two rats in each group. A portion of the tissue was fixed in 10% neutral formalin, embedded in paraffin, and stained with HE, Masson, and Pas staining to observe the morphological changes in kidney tissue and islet structure in the pancreas. The remaining renal tissue was stored at -80 ℃ for future analysis. Western blotting was performed to detect the expression of nephrin and podocin in renal tissue.

### Oxidative stress biochemical measurements in serum and kidney

The levels of malondialdehyde (MDA) and the activities of superoxide dismutase (SOD) and glutathione peroxidase (GSH) were measured in both serum and renal tissues using commercial assay kits (MDA, No. A003-1-2; SOD, No. A001-3-2; GSH, No. A006-2-1) obtained from Nanjing Jiancheng (Jiancheng, Nanjing, China).

### Cell culture

Human Kidney-2 (HK-2) cells were obtained from Professor Shen Lei (The First Affiliated Hospital of Soochow University, Suzhou, China) and cultured in low-glucose DMEM/F12 medium supplemented with 10% fetal bovine serum (FBS) and 1% penicillin/streptomycin (PS) at 37 ℃ in a 5% CO2 incubator.

### Cell viability assay

The MTT assay was used to evaluate cell viability. HK-2 cells (1×10^4^ cells/well) were seeded into a 96-well plate containing 100 μl of medium and incubated for 24 hours. The cells were divided into a blank group (without cells), a control group (without drugs), and different doses of NNAV (0.4, 0.8, 1.0, 1.6, and 2 μg/ml). Each group was replicated six times. After 24 and 48 hours of incubation, 20 µl of 5 mg/ml MTT was added to each well, followed by incubation for four hours. The supernatant was then removed and 150 µl of dimethyl sulfoxide was added to each well to stop the reaction. After shaking and mixing, the OD value at 570 nm was measured using a microplate reader (Bio-Rad550, Bio-Rad Laboratories, USA), and the inhibition rate was calculated as follows: Cell viability/%=(OD_NNAV_-OD_blank_) / (OD_control_-OD_blank_)×100. The IC50 was calculated based on the cell inhibition rate, which is the concentration of the drug that causes 50% cytotoxicity in the cells.

### Cell morphology and cell treatment

According to the literature and IC50 results from the MTT assay, doses of NNAV at 0.4 μg/ml, 0.8 μg/ml, and 1.0 μg/ml were used. Six groups were established: (1) Normal control group (NC, DMEM/F12); (2) High glucose group (HG, 30 mmol/L glucose + DMEM/F12); (3) Mannitol group (mannitol, 30 mmol/L D-mannitol + DMEM/F12); (4) High glucose + 0.4 μg/ml NNAV (HG+0.4 NNAV); (5) High glucose + 0.8 μg/ml NNAV (HG+0.8 NNAV); (6) High glucose + 1 μg/ml NNAV (HG+1 NNAV). The 30 mmol/L high glucose DMEM/F12 solution is hypertonic. To exclude the effect of hypertonicity on HK-2 cells, the mannitol group was included, and its osmotic pressure was consistent with that of the high glucose group. The morphology of HK-2 cells in each group was observed using an inverted phase-contrast microscope (Olympus, Japan).

### Immunofluorescence

Preparation of cell slides: HK-2 cells in the logarithmic growth phase were harvested and resuspended in DMEM/F12 culture solution containing 10% fetal bovine serum. The cell suspension was inoculated into a 6-well culture plate with sterile cover slides and cultured for 24 hours. After changing the culture medium according to the experimental groups, cells were cultured for an additional 24h and 48h. Control wells without drugs were included. The medium was discarded and cells were washed with PBS. The cells were fixed in 4% paraformaldehyde at room temperature for 30 minutes, air-dried, and washed with PBS. The cover glass was permeabilized with 0.1% TritonX-100 PBS at room temperature for 5 minutes, blocked with 10% goat serum BSA at 37 ℃ for 30 minutes, and incubated overnight at 4 ℃ with a-SMA and Collagen-I antibodies (dilution 1:100). After washing with PBS three times for five minutes each, FITC labeled goat anti-mouse IgG (dilution 1:200) was added and incubated at 37 ℃ for one hour. The cells were washed with PBS, stained with 1 μg/ml DAPI, and incubated at room temperature for 15 minutes. The cover glass was rinsed with double distilled water, sealed with 50% non-fluorescent buffered glycerin (diluted with PBS), and observed directly under a fluorescence microscope. Images were captured using a TCS SP2 fluorescence microscope. Negative controls were prepared by replacing the first antibody with PBS. Antibodies were visualized as green fluorescence and nuclei as blue fluorescence.

### Western blotting

Proteins were extracted from cells and kidney tissues using a protein extraction kit (Thermo, USA) following the manufacturer's instructions. The protein concentration of each sample was measured using the BCA method. SDS-PAGE (10%) was used to separate protein extracts (50 mg/lane). The proteins were then transferred onto PVDF membranes (Millipore, USA). The membranes were incubated overnight at 4 ℃ with primary antibodies, including E-cadherin (1:1000, Cell Signaling Technology, USA), TGF-β (1:1000, Cell Signaling Technology, USA), podocin (1:1000, Cell Signaling Technology, USA), and nephrin (1:1000, Cell Signaling Technology, USA). After washing, the membranes were incubated with the secondary antibody at room temperature for one hour. Proteins were detected by chemiluminescence, and the intensity of the bands was analyzed using Image J software.

### Statistical analysis

The data were expressed as mean ± SEM. Multigroup comparisons were performed using analysis of one-way ANOVA with SPSS 22.0. The data graph was created using GraphPad Prism 8.0 (GraphPad, San Diego, USA). A p-value less than 0.05 was considered statistically significant.

## Results

### General observation of rats

Diabetic mellitus (DM) rats exhibited symptoms such as polydipsia, polyuria, hyperphagia, weight loss, lethargy, and slow response. The bedding required frequent changes, and the urine had a noticeable smell of apples that had gone bad. After four weeks of DM induction, the rats developed a yellow coat color and crystal opacity. However, treatment with STZ+CTX (10, 20, 40μg/kg) did not result in significant improvement.

### Pathology changes after CTX treatment

**Figure 1.**
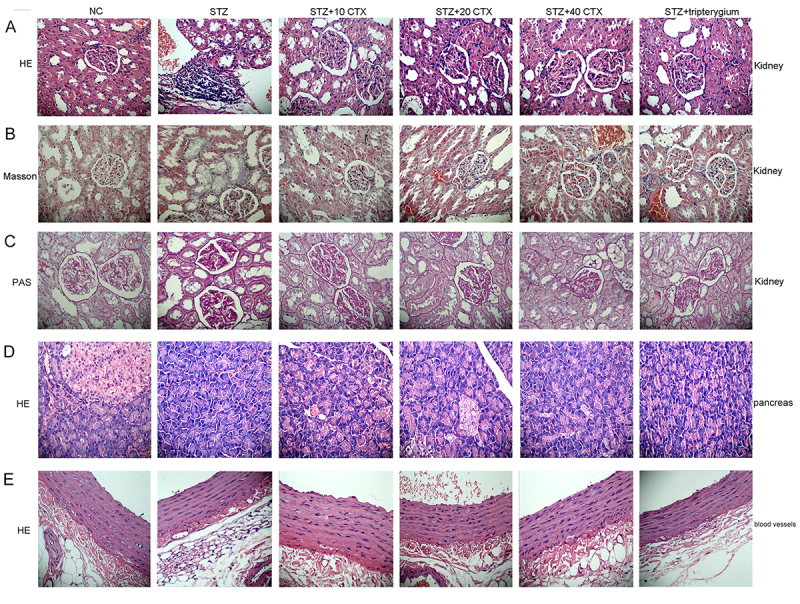
Pathological features of glomeruli, pancreas, and blood vessels in rats with DKD. **(A)** Hematoxylin and eosin staining of glomeruli. **(B)** Masson's trichrome staining of glomeruli. **(C)** Periodic acid-Schiff staining of glomeruli. **(D)** Hematoxylin and eosin staining of the pancreas. **(E)** Hematoxylin and eosin staining of blood vessels. Image magnification: 400x.

The pathological results of the kidney are presented in Figure 1A. Compared to the normal control group, the renal tubules in the STZ group showed vacuolar degeneration, narrowed lumen, and a large number of inflammatory cells infiltrating around, and no obvious abnormality was found under the pathological microscope of the glomerulus. Compared to the STZ group, the STZ+(10, 20, 40) CTX group, and STZ+tripterygium group also showed renal tubule injury, without significant recovery. Masson staining (Figure 1B) of the glomerulus is mainly used to evaluate the changes in glomerular fibrosis level. In this experiment, regardless of whether it was in the cortex or medulla of the glomerulus, the blue staining part in the model of DKD (STZ group) was significantly increased, suggesting that the expression of collagen fibers in the glomerulus and renal tubules was significantly increased. In the STZ+(10, 20, 40) CTX group and STZ+tripterygium group, the level of partial fibrosis could be reduced. However, the effect of neurotoxin and tripterygium on the treatment was not significant, and neither of them reached the degree of reversal. Glomerular PAS staining (Figure 1C) can be used to evaluate the morphological changes of the glomerulus. In the normal group, the structure of the glomerulus was intact without mesangial matrix hyperplasia. The STZ group showed glomerular structural disorder and proximal renal tubular injury. The drug intervention group (i.e. CTX and tripterygium group) could only alleviate this injury to a small extent, and there was no dose-dependent difference between cobra toxin. The pathological results of the pancreas are shown in Figure 1D. Compared to the normal control group, the islet structure of the model group was severely damaged, the islet volume was reduced and atrophied, and inflammatory cells infiltrated the islet cells. Tripterygium and CTX did not improve the condition after treatment. HE staining of blood vessels (Figure 1E) is used to evaluate the changes in blood vessels. In the normal control group, endothelial cells in the intima of blood vessels were arranged compactly, and smooth muscle cells and elastic fibers were seen in the middle membrane. In the STZ group, the elastic fiber structure disappeared, and the smooth muscle cells were damaged. After the intervention of drugs (i.e. CTX and tripterygium), a small number of elastic fibers can be seen in the middle membrane, but the structure is disordered. Although it has some improvement effect, it has not returned to normal.

### Influence on body weight, glucose, and urinary protein after CTX treatment

**Figure 2.**
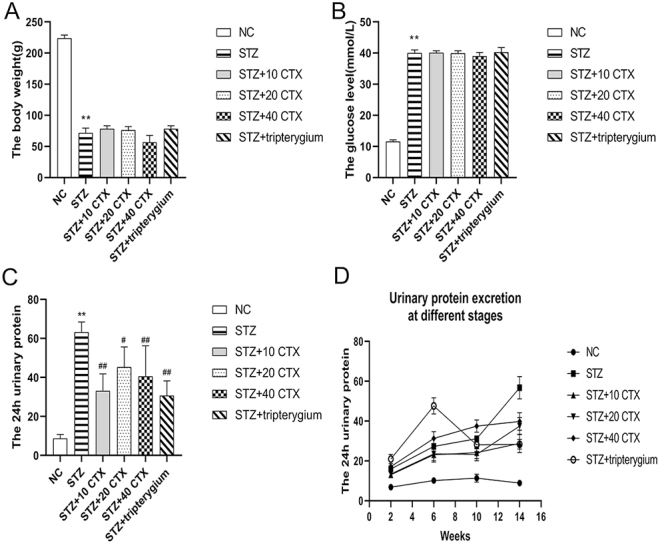
The effect of cobra toxin treatment on body weight, glucose, and urinary protein excretion in DKD. **(A)** Changes in body weight. **(B)** Changes in glucose levels. **(C)** 24-hour urinary protein excretion. **(D)** 24-hour urinary protein excretion in different stages. Group data are presented as mean ± SEM. * p < 0.05 and ** p < 0.01 indicate a significant difference compared with the normal control group; # p < 0.05 and ## p < 0.01 indicate a significant difference compared with the STZ group (n = 7).

There were significant differences in body weight (Figure 2A) and blood glucose (Figure 2A) between the normal control group and the STZ group (p < 0.01), while there were no significant differences between the STZ group and the drug treatment (CTX and tripterygium) groups (p > 0.05). Compared with the normal control group, the 24-hour urinary protein (Figure 2C) in the STZ group increased significantly (p < 0.01). Compared with the STZ group, 24-hour urinary protein decreased significantly after CTX and tripterygium treatment (p < 0.01). At the same time, we detected changes in 24-hour urinary protein at different stages (Figure 2D). The experimental results showed that compared with the STZ group, the urine protein in the tripterygium treatment group decreased significantly in the tenth week. The effect of CTX on protein reduction occurred in the 6th week, which was earlier than that of the tripterygium treatment group. With the extension of time, the protein-lowering effect of CTX gradually weakened.

### Influence on blood biochemical indexes after CTX treatment

**Figure 3.**
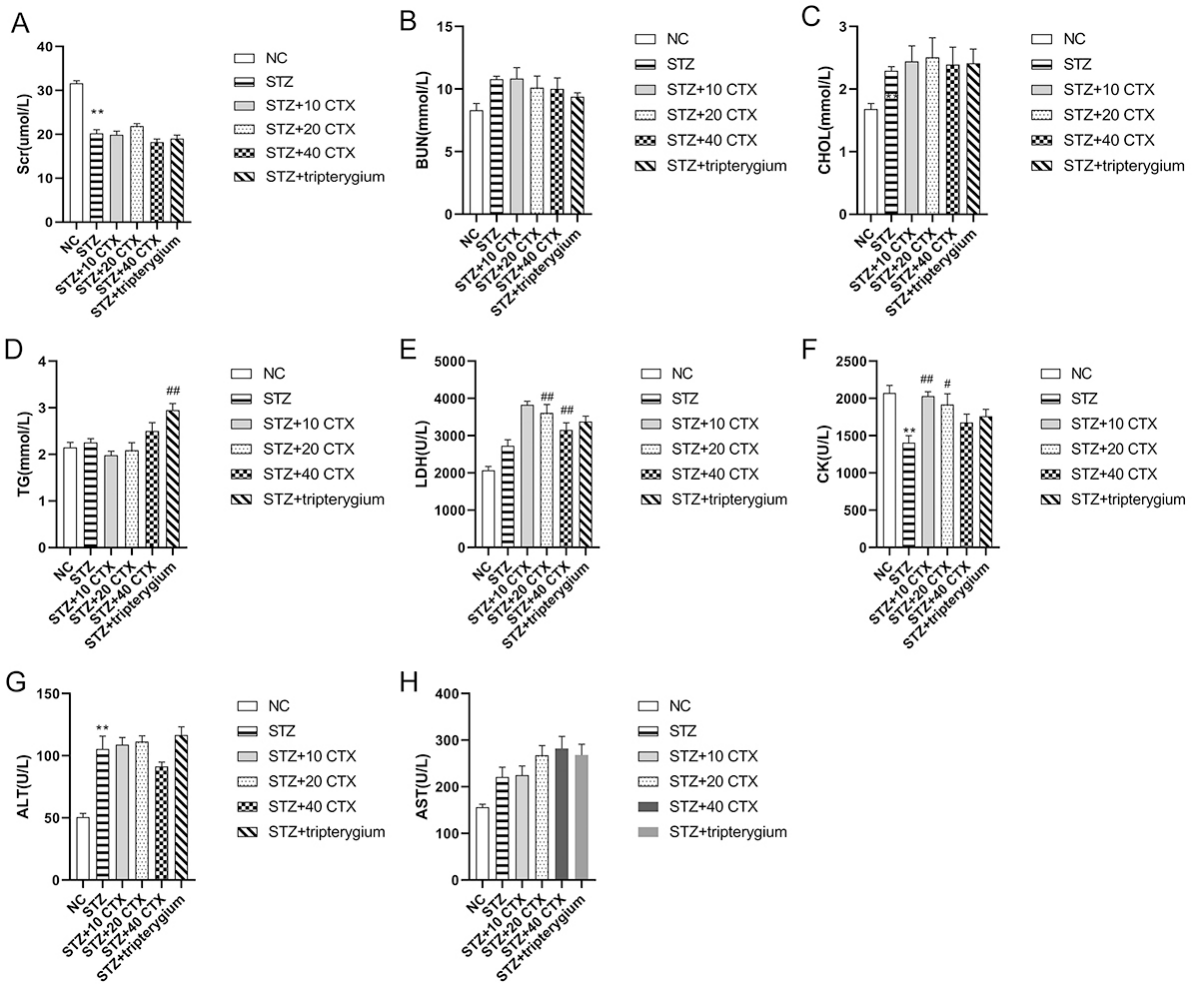
The impact of CTX Treatment on Blood Biochemical Indicators. **(A)** Plasma SCr. **(B)** Plasma BUN. **(C)** Plasma CHOL. **(D)** Plasma TG. **(E)** Plasma LDH. **(F)** Plasma CK. **(G)** Plasma ALT. **(H)** Plasma AST. Group data are expressed as mean ± SEM. * p < 0.05 and ** p < 0.01, significant difference compared with the normal control group; # p < 0.05 and ## p < 0.01, significant difference compared with the STZ group (n = 7).

Compared with the normal control group, there was a significant decrease in SCR (Figure 3A) in the STZ group (p < 0.01). However, there was no significant difference in SCR after treatment with CTX and tripterygium when compared to the STZ group. There were no significant differences observed in Bun (Figure 3B), CHOL (Figure 3C), and AST (Figure 3H) among the groups. In addition, there was no significant difference in TG (Figure 3D) between the normal control group and the STZ group. However, after tripterygium treatment, there was a significant increase in TG levels (p < 0.01) when compared to the STZ group. No significant difference was observed in LDH (Figure 3E) between the normal control group and the STZ group. Nevertheless, a significant decrease in LDH was observed in STZ+(20, 40) CTX (p < 0.01) when compared to the STZ group. In comparison to the STZ group, there was a significant decrease in CK (Figure 3F) in the normal control group (p < 0.01). Conversely, after CTX treatment, there was a significant increase in CK levels (p < 0.05 or p < 0.01). Furthermore, ALT (Figure 3G) was significantly higher in the STZ group than in the normal control group (p < 0.01). However, after treatment with CTX and tripterygium, there was no significant change observed when compared to the STZ group.

### Oxidative stress changes after CTX treatment

**Figure 4.**
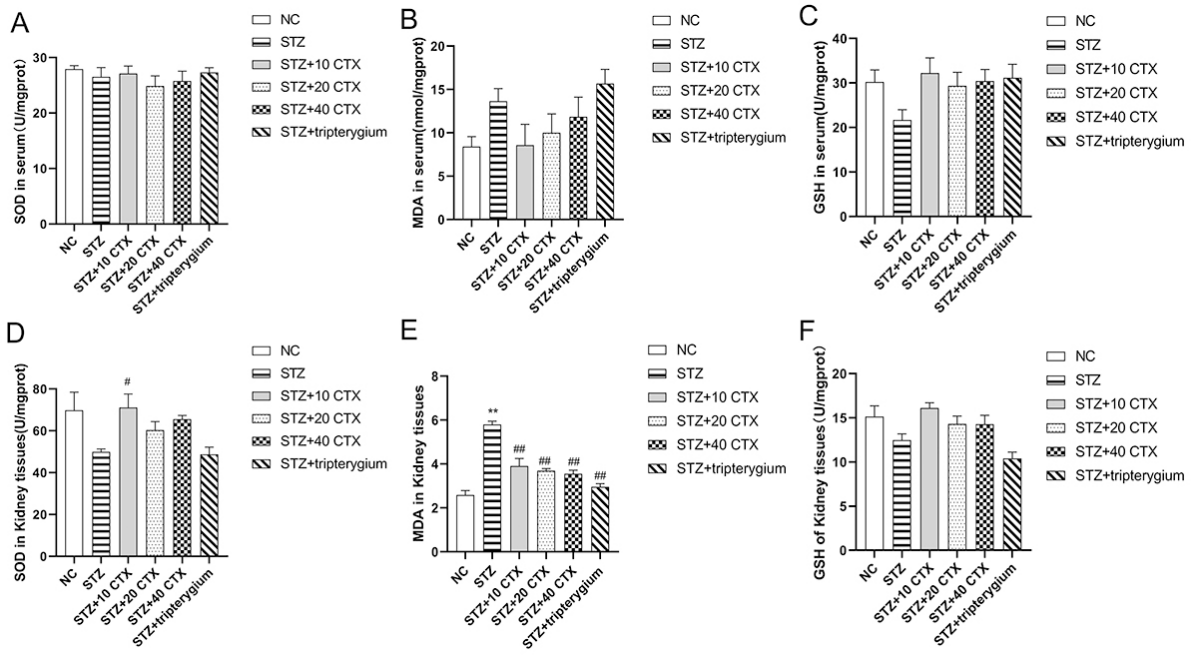
The impact of treatment of CTX on oxidative stress. **(A)** SOD in serum. **(B)** MDA concentrations in serum. **(C)** GSH concentrations in serum. **(D)** SOD in kidney tissues. **(E)** MDA in kidney tissues. **(F)** GSH in kidney tissues. Group data were expressed as mean ± SEM. * p < 0.05 and ** p < 0.01 , significant difference compared with the normal control group; # p < 0.05 and ## p < 0.01 , significant difference compared with the STZ group (n = 7).

The impact of CTX treatment on oxidative stress was assessed by examining changes in levels of ROS, GSH, and MDA (Figure 4). No significant differences were observed between groups in serum SOD, MDA, and GSH levels (Figure 4A, B, C). In comparison with the normal control group, the expression of SOD (Figure 4D) and GSH (Figure. 4F) in renal tissue of the STZ group decreased, while the expression of MDA (Figure 4E) increased, although no significant differences were noted. Following treatment with CTX, the expression of SOD (Figure 4D) in renal tissue of the STZ+10 CTX group significantly increased (p < 0.05). The expression of MDA (Figure 4E) in renal tissue of the STZ group also significantly increased (p < 0.01). However, after treatment with both CTX and tripterygium, the expression of MDA decreased significantly (p < 0.01). No significant difference in GSH (Figure 4F) expression was observed among the groups.

### The expression of podocin and nephrin changes after CTX treatment

**Figure 5.**
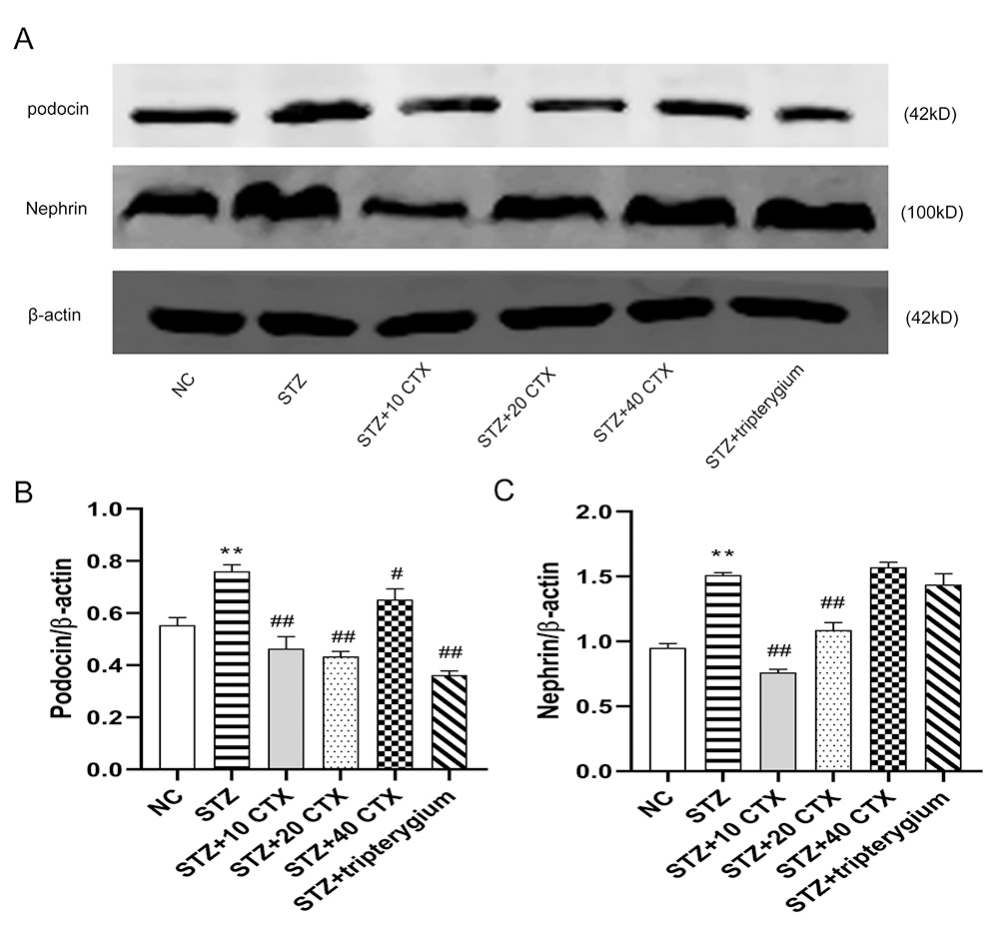
CTX significantly relieves the expression of both podocin and nephrin in renal tissues. **(A)** Western blot analysis for the protein levels of podocin and nephrin in renal tissues. **(B)** The quantitation of signal intensities was analyzed by using Image J software and shown after being normalized by the housekeeping β-actin. Group data were expressed as mean ± SEM. * p < 0.05 and ** p < 0.01, a significant difference compared with the normal control group; # p < 0.05 and ## p < 0.01, a significant difference compared with the STZ group (n = 3). **(C)** The nephrin quantitation of signal intensities was analyzed by using Image J software and shown after being normalized by the housekeeping β-actin.

In this study, the authors aimed to evaluate the effects of CTX treatment on the expression of podocin and nephrin in the context of STZ-induced nephropathy. They studied the changes in the expression of podocin and nephrin levels using Figure 5 . Compared to the normal control group, the expression of podocin protein (Figure 5B) in the STZ group increased significantly (p < 0.01). In contrast, the expression of podocin protein in the STZ+(10, 20, 40) CTX groups and STZ+tripterygium groups decreased and tended to be normal, showing a statistically significant difference (p < 0.05 or p < 0.01) when compared to the STZ group. Similarly, the expression of nephrin protein (Figure 5C) was significantly increased in the STZ group compared to the normal control group (p < 0.01). Conversely, the expression of nephrin protein in the STZ+(10, 20) CTX groups decreased and tended towards normalization, exhibiting a statistically significant difference (p < 0.01) compared to the STZ group. However, there was no difference observed between the STZ+tripterygium group and the STZ group. Taken together, these findings suggest that CTX treatment may have a favorable effect on podocin and nephrin expression in STZ-induced nephropathy. In contrast, tripterygium treatment may not have a significant impact on nephrin expression. Further research is warranted to elucidate the therapeutic potential of CTX in the management of nephropathy.

### Effects of NNAV on HK-2 Cells in a High-Sugar Environment

**Figure 6.**
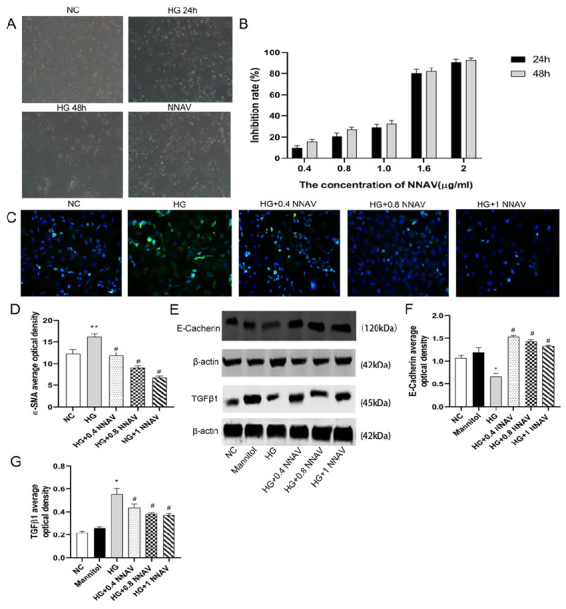
NNAV significantly relives transdifferentiation of renal tubular epithelial cells in a high glucose environment. **(A)** Cell morphology. **(B)** Inhibition rate of NNAV on HK-2 cells. **(C)** α-SMA immunofluorescence staining. Image magnification×400. **(D)** α-SMA average optical density. **(E)** Western blot analysis for the protein levels of E-cadherin and TGF-β1 in HK-2. (F) E-cadherin average optical density. **(G)** TGF-β1 average optical density. Group data were expressed as mean ± SEM. * p < 0.05 and ** p < 0.01, significant difference compared with the normal control group; # p < 0.05 and ## p < 0.01, significant difference compared with the STZ group (n = 3).

The cell morphology of each group was observed using an inverted phase contrast microscope (Figure 6A). Normal HK-2 cells are round or polygonal adherent cells, closely connected, and monolayer fused into a paving stone-like arrangement. After 24 hours of high glucose stimulation, the cell morphology did not change significantly. After 48 hours of stimulation with high glucose, some cells changed shape, stretched and elongated, and became spindle-shaped. After 48 hours of NNAV intervention, there was no significant difference between cell morphology and the normal group. The MTT method was used to calculate the inhibition rates of NNAV 0.4, 0.8, 1.0, 1.6, and 2 μg/ml for 24 and 48 hours after intervention on HK-2 cells (Figure 6B). When the NNAV concentration was the same, the inhibition rate at 48 hours was higher than that at 24 hours. Compared with the normal control group, the inhibition rate of HK-2 cells increased with the increase of NNAV concentration, which was positively correlated with the drug concentration (r = 0.937, p < 0.01). The linear regression equation calculated that the IC50 was 1.4 μg/ml. Therefore, 0.4, 0.8, and 1.0 μg/ml concentrations of NNAV were used in this experiment to interfere with HK-2 cells. The results of immunofluorescence staining (Figure 6C, D) showed that the expression of α-SMA significantly increased after 48 hours of high glucose intervention compared with the normal control group (p < 0.01). After intervention with different concentrations of NNAV in a high glucose environment, the expression of α-SMA decreased significantly in a concentration-dependent manner (p < 0.05). Western blot (Figure 6E, F, G) showed that there was no significant difference in the expression of E-cadherin and TGF-β1 between the mannitol group and the normal control group. After high glucose intervention, compared with the normal control group, the expression of E-cadherin was significantly reduced, and the expression of TGF-β1 was significantly increased (p < 0.05). After 48 hours of intervention with different concentrations of NNAV (0.4, 0.8, 1.0 μg/ml), the expression level of E-cadherin was significantly higher than that of the high glucose group, while the expression level of TGF-β1 was significantly lower than that of the high glucose group (P<0.05).

## Discussion

Proteinuria is a critical clinical manifestation of DKD, with long-term albuminuria being an independent risk factor for the progression of renal function impairment in chronic kidney disease [18]. Treatment aimed at reducing urinary protein excretion can delay the progression of nephropathy. The production of albuminuria is closely related to the destruction of the normal structure of the glomerular filtration barrier or its functional damage [19]. The glomerular filtration membrane comprises visceral epithelial cells, basement membrane, and endothelial cells [20]. The visceral epithelial cells, or podocytes, are located outside the basement membrane and form the final barrier to prevent protein loss [21]. The structure of podocytes is divided into three parts: cell body, primary process, and foot process [22]. The 30-40nm holes between adjacent foot processes are connected by nephrin, podocin, CD2-related protein (CD2AP), FAT, ZO-1, and other protein molecules, thus preventing the passage of macromolecular substances and making the glomerular filtration membrane highly selective for the permeability of substances in plasma [23]. In this study, after 14 weeks of modeling, the expression of serum creatinine in rats decreased. Combined with the pathological results, some glomerular compensatory hypertrophy indicates that they are in the early compensatory proliferation stage of diabetes nephropathy. The renal tubules of MN mice exhibit vacuolar degeneration, narrowed lumen, a large number of inflammatory cells infiltrating around, and a significant increase in glomerular volume. The expression of collagen fibers in the glomerulus and renal tubules significantly increases, and the mesangial matrix proliferates. These pathological characteristics suggest that MN mice have compensatory glomerular hypertrophy and are in the early compensatory proliferation stage of diabetes nephropathy. At the 14th week, the model group had developed microalbuminuria, while the tripterygium and low-dose CTX intervention groups had not yet reached the standard of microalbuminuria, indicating that tripterygium and low-dose CTX can reduce the excretion of proteinuria. However, the period for reducing proteinuria was different between tripterygium and low-dose CTX. After the treatment of tripterygium and low-dose CTX, the 24-hour urine protein of diabetes nephropathy mice decreased significantly. After 10 weeks, the urinary protein of tripterygium significantly decreased, while the low-dose CTX showed the most significant reduction in urinary protein at 6 weeks. Nephrin and podocin proteins have changed in the early compensatory and proliferative stage of diabetes nephropathy, while tripterygium and CTX can interfere with this change in the early stage to make it close to the normal level, which may be one of the mechanisms of its early protein reduction.

The pathogenesis of diabetes nephropathy is the result of multiple factors. In the case of diabetes, the production of reactive oxygen species is often increased or the function of the antioxidant enzyme system SOD and GSH is reduced, which breaks the balance between the production and clearance of local reactive oxygen species in renal tissue [24]. SOD is an antioxidant enzyme that disproportionates superoxide anion free radicals in the body. The level of SOD in serum can reflect the neutralization and scavenging capacity of free radicals in the body [25]. Malondialdehyde (MDA) is the most common lipid peroxidation product. The amount of MDA can measure the degree of lipid peroxidation and indirectly reflect the level of active oxygen produced by lipid oxidation [26]. The metabolic disorder of oxygen free radicals often occurs in the early stage of diabetes before the morphological change of the kidney. Oxygen free radicals may damage the basement membrane and vascular endothelial cell membrane or may participate in the change process of glomerular hemodynamics in the early stage of diabetes, ultimately leading to renal dysfunction [27]. In this study, compared with the STZ group, the NNAV intervention group had an overall increase in GSH, SOD and a decrease in MDA, which had an antioxidant effect. In this study, after NNAV treatment, there was no hypoglycemic effect and no islet recovery, but there was a protein-lowering effect.

Currently, it is believed that renal tubulointerstitial fibrosis (RIF) represents a common pathway and the primary pathological basis for various chronic kidney diseases, including DKD, leading to end-stage renal disease [28]. The fundamental pathological characteristic of renal interstitial fibrosis is the excessive accumulation of extracellular matrix protein (ECM) in the renal interstitium [29]. The accumulation of MyoF in the renal interstitium is considered the key factor of renal tubulointerstitial fibrosis [30]. Transdifferentiation of renal tubular epithelial cells into myofibroblasts (TEMT) refers to this process, and α-SMA serves as a phenotypic marker for the expression of MyoF in renal tubular epithelial cells [31]. E-cadherin, a calcium-dependent transmembrane glycoprotein, is an essential marker that reflects the features of epithelial cells, mediates the adhesion of homogeneous cells to each other, and plays a crucial role in maintaining cell integrity and polarity [32].

In this study, after human proximal renal tubular epithelial cells were exposed to high glucose for 48 hours, phase contrast microscopy indicated that some epithelial cells changed their morphology, transitioning from the original oval or polygonal shape to spindle-shaped, myofibroblast-like cells. These morphological changes suggest that some HK-2 cells might undergo transdifferentiation into MyoF. To investigate whether TEMT occurred in HK-2 cells after 48 hours of high glucose exposure, Western blotting was employed to detect the expression of E-cadherin. Immunofluorescence revealed an increased expression of α-SMA, indicating that human proximal renal tubular epithelial cells exposed to a high glucose environment underwent transdifferentiation. To eliminate the possibility that high osmotic pressure from high glucose-induced the TEMT process, a mannitol group was established, which maintained the same osmotic pressure as the high glucose group. Phase contrast microscopy revealed that cell morphology was normal, and the expression of E-cadherin, α-SMA, and the corresponding protein expression of the normal group had no statistical significance. Thus, it could be concluded that high osmotic pressure did not induce transdifferentiation of HK-2 cells. TGF-β1 is considered a key factor leading to fibrosis, a classical regulator, and an essential inducer of tubular epithelial cell transdifferentiation [33]. It is closely related to the occurrence and development of DKD, as it promotes the synthesis of ECM components such as collagen, fibronectin, laminin, and proteoglycan, inhibits the synthesis of enzymes that degrade ECM components, and induces its formation through autocrine action, significantly enhancing its biological activity, exerting its effect of promoting fibrosis, and ultimately causing renal interstitial fibrosis [34]. 

## Conclusion

The findings of this study suggest that high glucose can induce the transdifferentiation of HK-2 cells to MyoF, leading to tubulointerstitial fibrosis. This process is characterized by decreased E-cadherin expression and increased expression of α-SMA and TGF-β1. However, treatment with NNAV can inhibit this transdifferentiation and reduce the expression of TGF-β1, which may be a potential mechanism for its preventative effects against DKD. Furthermore, CTX has demonstrated an antioxidant effect in diabetes model rats and can reduce urine protein levels at an early stage in a dose-independent and time-dependent manner. This early reduction in protein may be attributed to CTX's ability to regulate the expression of nephrin and podocin proteins. Overall, these results suggest that NNAV and CTX may have therapeutic potential for the prevention and treatment of DKD.

### Abbreviations

DM: Diabetes mellitus; DKD: Diabetic kidney disease; STZ: Streptozotocin; NNAV: Naja naja arta venom; Glu: Glucose; BUN: Blood Urea Nitrogen; SCr: Serum Creatinine; PRO-U: Urinary protein; MDA: Malondialdehyde; SOD: Superoxide Dismutase; TC: Total cholesterol; TG: Triglyceride; TGF-β1: Transformation Growth Factor-β1; α-SMA: α-smooth muscle actin; ECM: extracellular matrix protein; HK-2: human proximal tubular epithelial cells; MyoF: myofibroblast; TIF: tubulointerstitial fibrosis; TEMT: tubular Epithelial myofibroblast transdifferentiation; WB: western-blotting; CTX: cobra α-neurotoxin.
